# Establishment of a gnotobiotic pig model of *Clostridioides difficile* infection and disease

**DOI:** 10.1186/s13099-022-00496-y

**Published:** 2022-06-06

**Authors:** Charlotte Nyblade, Viviana Parreno, Peng Zhou, Casey Hensley, Vanessa Oakes, Hassan M. Mahsoub, Kelsey Kiley, Maggie Frazier, Annie Frazier, Yongrong Zhang, Hanping Feng, Lijuan Yuan

**Affiliations:** 1 Department of Biomedical Sciences and Pathobiology, Virginia-Maryland College of Veterinary Medicine, Virginia Polytechnic Institute and State University, Blacksburg, VA 24061 USA; 2 Department of Microbial Pathogenesis, University of Maryland at Baltimore, Baltimore, MD 21201 USA; 3 INCUINTA, Instituto Nacional de Tecnologia Agropecuaria (INTA), Instituto de Virologia e Innovaciones Tecnologicas (IVIT INTA CONICET), Buenos Aires, Argentina; 4 Center for Emerging, Zoonotic, and Arthropod-Borne Pathogens, Virginia Polytechnic Institute and State University, Blacksburg, VA 24061 USA

**Keywords:** *Clostridioides difficile* infection/illness (CDI), Gnotobiotic pigs, Pseudomembranous colitis (PMC)

## Abstract

*Clostridioides difficile* (*C. difficile*) is a gram-positive, spore-forming, anaerobic bacterium known to be the most common cause of hospital-acquired and antibiotic-associated diarrhea. *C. difficile* infection rates are on the rise worldwide and treatment options are limited, indicating a clear need for novel therapeutics. Gnotobiotic piglets are an excellent model to reproduce the acute pseudomembranous colitis (PMC) caused by *C. difficile* due to their physiological similarities to humans and high susceptibility to infection. Here, we established a gnotobiotic pig model of *C. difficile* infection and disease using a hypervirulent strain. *C. difficile*-infected pigs displayed classic signs of *C. difficile* infection, including severe diarrhea and weight loss. Inoculated pigs had severe gross and microscopic intestinal lesions. *C. difficile* infection caused an increase in pro-inflammatory cytokines in samples of serum, large intestinal contents, and pleural effusion. *C. difficile* spores and toxins were detected in the feces of inoculated animals as tested by anaerobic culture and cytotoxicity assays. Successful establishment of this model is key for future work as therapeutics can be evaluated in an environment that accurately mimics what happens in humans. The model is especially suitable for evaluating potential prophylactics and therapeutics, including vaccines and passive immune strategies.

## Background

*Clostridioides difficile* (*C. difficile*) is a gram-positive, spore-forming, anaerobic bacterium that is the most common cause of hospital-acquired and antibiotic-associated diarrhea [1]. *C. difficile* colonization of the colon results in a wide range of clinical presentations ranging from asymptomatic carriage, diarrhea of varying severity, toxic megacolon, and life-threatening fulminant pseudomembranous colitis (PMC) [2]. Variation in symptom presentation is due to toxin production and underlying risk factors. Toxigenic strains of *C. difficile* release two enterotoxins, toxin A (TcdA) and toxin B (TcdB), which cause damage to the host intestinal cell through the inactivation of Rho and Ras GTPases; this damage triggers the host inflammatory response, causing an influx of cytokines and neutrophils that exacerbate intestinal damage [3]. In some strains, a binary toxin (CDT) is also produced [4]. Development of systemic disease is associated with toxemia [5, 6], however, the relative roles of all three toxins in causing local and systemic disease require further study [7, 8]. Underlying risk factors such as age, prior antibiotic treatment, immune-comprised health status, and/or prior *C. difficile* infection all also influence symptom severity [1]. Populations ≥ 65 years of age are at a significantly higher risk of both contracting *C. difficile* and experiencing severe illness [9]. The use of broad-spectrum antibiotics disturbs the native intestinal microbiota, eliminating competing microbes and enabling *C. difficile* overgrowth in the colon [1].

Current treatment options for CDI are complicated by the persistence of *C. difficile* spores and the emergence of antibiotic-resistant strains [10]. It is therefore a high priority to develop a means of preventing CDI to circumvent the complications associated with treatment. Testing such a therapeutic requires a model that accurately represents the progression of CDI in humans. Piglets are ideal candidates as their digestive and immune systems are physiologically similar to humans. Furthermore, early studies investigating CDI in piglets indicate that they develop clinical signs and intestinal lesions which mimic those seen in humans [11–13]. The possibility of a gnotobiotic (Gn) piglet model for CDI is highly attractive, as Gn piglets have a microbiota-free gut and they are highly susceptible to infection [13]. The goal of this study was to establish a Gn pig model of CDI for future use in testing a novel therapeutic.

## Methods

### Clostridioides difficile inoculum

Spores of the *C. difficile* UK1 (027/B1/NAP1) hypervirulence strain were prepared as previously described [14]. Briefly, the UK1 vegetative cells were incubated in brain heart infusion-supplemented (BHIS) medium in 6-well plates anaerobically for 4 to 7 days to induce sporulation. The spores and residual vegetative cells were washed off the 6-well plates with sterile water. The pellet was collected by centrifugation for 1 min at 14,000×*g* and washed with ice-cold sterile water 3 times. The pellet was then resuspended in sterile water and heated at 60 °C for 20 min to kill vegetative cells. The spores were numerated by spreading serially diluted suspension onto BHIS agar plates containing 0.1% taurocholate.

### Animals and study design

**Fig. 1:**
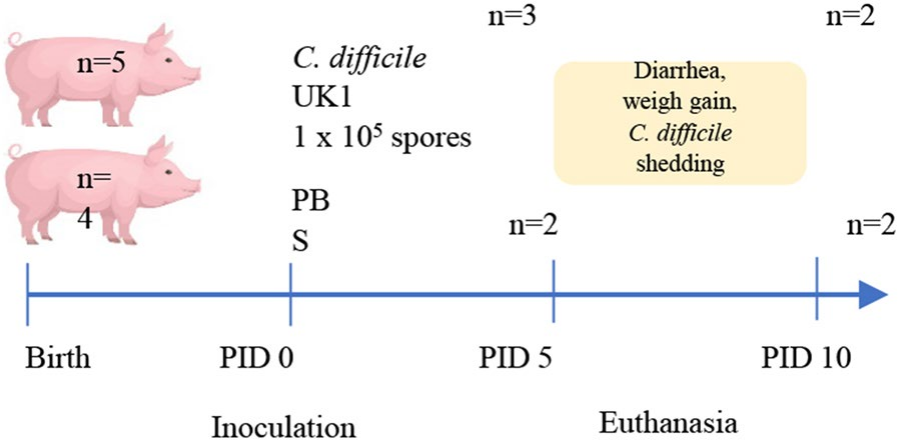
Study timeline. At 5 days of age (PID0), 5 gnotobiotic pigs received 1 × 10^5^ spores of *C. difficile* while 4 pigs received PBS as a negative control. Throughout the study timeline diarrhea scores, weight, and rectal swabs were obtained daily. At PID5, 3 pigs from the *C. difficile* group and 2 from the PBS group were euthanized. The remaining pigs from each group were euthanized at PID10

Gn pigs, derived from surgical hysterectomy, were housed inside sterile isolators and fed commercial ultra-high temperature (UHT), sterile-cow milk as previously described [13]. To test for sterility, 2 rectal swabs were collected from all pigs in isolators three days after birth. One swab was streaked on blood agar plates (for aerobic bacteria) and the second swab was swirled in 8 ml of thioglycolate broth (for anaerobic bacteria). Both plates and broth were incubated at 37 °C and monitored for 72 h. At 5 days of age, five animals were orally inoculated with 1 × 10^5^ spores of the *C. difficile* UK1 strain suspended in 5 ml of sterile water while the remaining four animals received 5 ml PBS as a negative control. Clinical signs of infection such as diarrhea, dehydration, weakness, lethargy, anorexia, and dyspnea were monitored and registered throughout the experiment. Rectal swabs, diarrhea scores, and weight were collected daily. On post-inoculation day 5 (PID 5), two pigs from the negative control group and three from the *C. difficile* group were euthanized. The remaining piglets from both groups were euthanized at PID10. Euthanasia time points were selected to monitor the development of acute (PID 5) and systemic diseases (PID 10). Blood was collected via jugular venipuncture prior to euthanasia. Following euthanasia, sections of the cecum and colon were collected and fixed in formalin for histology analysis. Additionally, large intestinal contents (LIC) and pleural effusion (if present) were collected for evaluating *C. difficile* counts, toxins, and pro-inflammation cytokines. Gross gastrointestinal and systemic lesions were noted during necropsy. The study timeline and division of Gn pigs into experimental groups are summarized in Fig. 1 and Table 1 respectively.

**Table 1:** Division of Gn pigs into experimental groups

# of pigs and euthanasia day	Pig ID	Inoculum
2 (PID 5)	Gp4-1-2021	PBS
	Gp4-2-2021	
2 (PID 10)	Gp4-3-2021	
	Gp4-4-2021	
3 (PID 5)	Gp4-5-2021	1 × 10^5^ PFU *C. difficile* UK1
	Gp4-6-2021	
	Gp4-7-2021	
2 (PID 10)	Gp4-8-2021	
	Gp4-9-2021	

### Diarrhea scoring

Fecal swabs were collected daily to determine diarrhea severity. Severity was determined based on the appearance of the feces using a scoring system of 0–3 (0 = solid, 1 = pasty, 2 = semisolid, 3 = liquid). A score of ≥ 2 indicated diarrhea. Statistical significance was determined with Students T-test. To test the significance of diarrhea onset days, negative control pigs were assigned an onset day of 11 (since the experiment ended on PID 10).

### Weight evolution

All Gn pigs were weighed daily from PID 0 until euthanasia. Weight evolution was plotted as a percentage of weight gain relative to the animal’s initial weight on PID 0. Statistical significance was not calculated due to the small number of experimental groups.

### Gross and microscopic evaluation of tissue

The large intestinal tissues (colon and cecum) were examined in situ before removal from the body cavity. Tissues were examined for fluid accumulation (edema), inflammation, dilation, and other lesions. The presence of pleural effusion was determined by using a needle to puncture the lung cavity and aspirating any fluid present. Sections of the colon and cecum were collected in 4% formaldehyde and submitted to the Virginia Tech Animal Laboratory Services (ViTALS) at Virginia-Maryland College of Veterinary Medicine for H&E staining and examination of histopathological changes and inflammation. A board-certified veterinary pathologist who was blinded with the pigs’ infection status examined the slides and provided the report.

### Cytokine assay

Blood samples were incubated for 1 h at 37 °C, incubated overnight at 4 °C, and then centrifuged at 1026×*g* for 15 min to separate serum. Serum was removed and heat-inactivated for 30 min at 56 °C. Intestinal contents and pleural effusion samples were centrifuged at 2236×*g* for 30 min to remove any *C. difficile* particles and then filtered by a 0.22 μm centrifugal device (Millipore, cat no. UFC40GV0S). The absence of *C. difficile* spores was confirmed by streaking samples on BRU, LKV, and PEA agar plates, placing all plates in an inflatable anaerobic bag with an anaerobic indicator strip, and incubating at 35 °C for 3 days. Anaerobic culture was performed by the ViTALS at Virginia-Maryland College of Veterinary Medicine. All samples were then shipped to Eve Technologies in Calgary, Canada where a Porcine Cytokine 13-Plex Discovery Assay was used to measure concentrations of pro-inflammatory cytokines. Samples were tested in duplicate and an average concentration of the respective cytokine was determined. In some samples, the cytokine concentration was out of range, in which case a value was extrapolated based upon the sample’s observed fluorescence intensity relative to a standard curve’s fluorescence intensity. To determine the fold change in a cytokine, the average concentration for each experimental group was determined, and then the *C. difficile* average was divided by the negative control average. Statistical significance was determined by Student’s T-test. Because pleural effusion was only present and collected from two *C. difficile* infected pigs, the cytokine concentrations of the individual pigs (Gp4-8-21 and Gp4-9-21) were presented rather than the fold change.

### Clostridioides difficile colony counting

The cotton part of each rectal swab stick was cut off and soaked in 600 µl of sterile PBS in a 1.5 ml Eppendorf tube on ice for at least 30 min. Tubes were vortexed to dissolve excretion on cotton into the PBS. Supernatants of samples were serially diluted 10 times, and 5 µl of each sample was loaded onto chromogenic (CHROMIDTM, cat. no 43871) plates. Plates were left in COY anaerobic chamber for 24–48 h to identify *C. difficile.* After 24–48 h, plates were removed from the chamber and colonies were counted. Any numbers within 0–60 range were considered as countable. Too many too count (TMTC) means that even after a 10^6^-time dilution, the colonies of the 5 µl grown on agar plate were more than 60.

### Clostridioides difficile cytotoxicity assay

The cotton part of each rectal swab stick was soaked in 600 µl of PBS, vortexed, incubated on ice for 1 h, and vortexed again. Samples were diluted 1 to 10 in media and centrifuged at 20,000×*g* for 10 min at 4 °C. The supernatant was added to dilution plates and serially diluted. Dilutions were added directly to Vero cells plated at a concentration of 1.25 × 10^4^ cells/well in 96 well plates. Each sample was tested in duplicate with 2 control wells. Control wells also received 10 µl of the neutralizing antibody FZ003 (concentration of 1 µg/ml) to validate that cell rounding was due to *C. difficile* toxins. Plates were incubated overnight at 37 °C with 5% CO_2_. Following incubation, the percentage of cell rounding in each well was calculated and the titer was determined as the highest dilution to cause 100% cell rounding.

## Results

### Clinical signs of infection

**Fig. 2:**
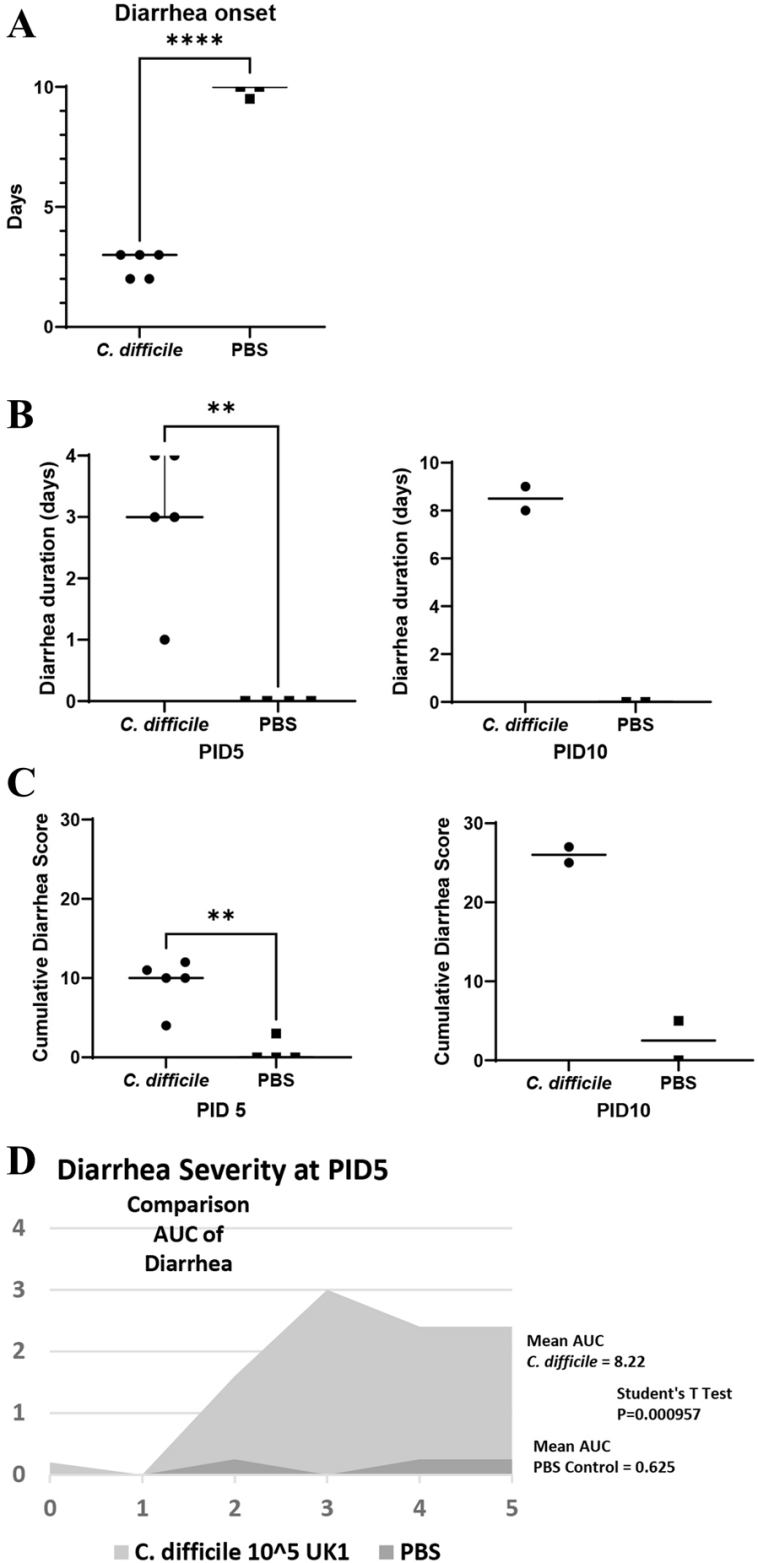
Diarrhea onset, duration, and severity in Gn pigs. *C. difficile* infected pigs developed diarrhea within 48–72 h of inoculation. Student’s T-test, p < 0.0001 (**A**). All *C. difficile* inoculated pigs had diarrhea for some length of time while none of the PBS control pigs developed diarrhea at any point in the experiment. Student’s T-Test, p < 0.01 (**B**). At PID 5 and PID 10, *C. difficile* inoculated pigs had significantly higher cumulative diarrhea scores than PBS control pigs. Student’s T-test, p < 0.01 (**C**). The area under the curve (AUC) of diarrhea in *C. difficile* infected pigs was significantly higher than the PBS control pigs. Student’s T-test p < 0.001 (**D**)

**Fig. 3:**
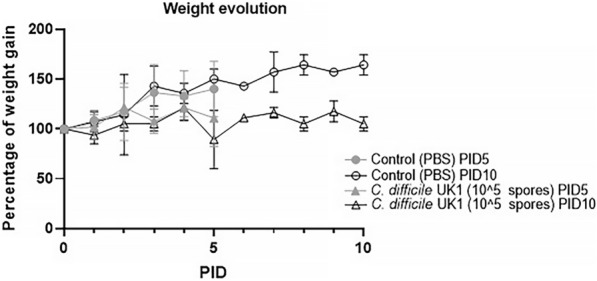
Weight changes in pigs. For both the PID 5 and PID 10 timepoints, PBS control pigs had a higher percentage of weight gain than their *C. difficile* inoculated counterparts

A complete summary of clinical signs between groups can be found in Table 2. Throughout the study, none of the PBS control pigs developed diarrhea. In comparison, all pigs inoculated with *C. difficile* developed diarrhea within 48–72 h of inoculation (Fig. 2A). Diarrhea consistency followed the typical CDI progression from yellow–brown and pasty to yellow and watery and was persistent for some length of time in all *C. difficile*-infected pigs (Fig. 2B). On average, *C. difficile*-infected pigs in the PID 5 group had diarrhea for 3 days while *C. difficile*-infected pigs in the PID 10 group had diarrhea for 8.5 days. The severity of diarrhea as indicated by the cumulative diarrhea score is shown in Fig. 2C. *C. difficile-*infected pigs had significantly higher mean cumulative diarrhea scores than PBS control pigs at both timepoints. At PID 5, the mean cumulative diarrhea score was 0.75 for PBS control pigs and 9.4 for *C. difficile-*infected pigs. At PID 10, the scores were 2.5 for the control pigs and 26 for *C. difficile-*infected pigs. The area under the curve (AUC) of diarrhea in *C. difficile*-infected pigs (8.22) was significantly higher than PBS control pigs (0.625) (Fig. 2D). *C. difficile-*infected pigs had a lower total weight-gain (0.1 kg at PID 5 and PID 10) than PBS control pigs (0.5 kg at PID 5 and 0.9 kg at PID 10) as well as a lower mean daily weight gain (Fig. 3).

**Table 2:** Summary of clinical signs of Gn pigs during PID0–5 and PID 0–10

Treatment group	n	% affected (%)	Diarrhea onset (mean days)	Diarrhea duration (mean days PID 0–5)	Mean cumulative diarrhea score PID 0–5	Mean daily weight gain from PID 0–5	Total weight gain from PID 0–5^a^	Average weight at PID 5 (kg)	n	Mean days with diarrhea PID 0–10	Mean cumulative diarrhea score PID 0–10	Mean daily weight gain from PID 0–10	Total weight gain from PID 0–10	Average weight at PID 10 (kg)
*C. difficile* infected	5	100	2.6 (0.24)^b^	3.0 (0.55)^Ac^	9.4 (1.4)^A^	0.02 (0.10)^A^	0.1	1.6	2	8.5 (0.35)^A^	26 (0.71)^A^	0.09 (0.05)^A^	0.1	1.9
Mock-infected control	4	0	N/A	0 (0)^B^	0.75 (0.75)^B^	0.10 (0.05)^A^	0.5	2	2	0 (0)^B^	2.5 (1.77)^B^	0.01 (0.09)^B^	0.9	2.3

^a^Total weight gain is the sum of mean daily weight gain in each treatment group. All data for PID 0–5 included pigs euthanized on PID 5 and PID 10

^b^Number in parenthesis is standard error of the mean

^c^Different capital letters (A, B) indicate significant difference among treatment groups for the same time point while shared letters indicate no significant difference. For diarrhea data, significance was determined with Student’s T test p < 0.05. For weight data, significance was determined with 2 Way ANOVA p < 0.05

### Morphological changes in tissue

**Fig. 4:**
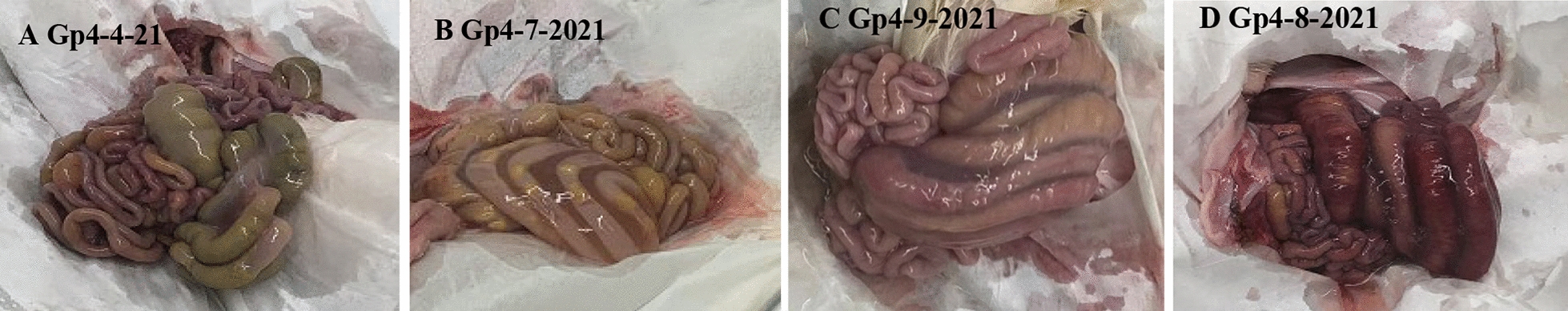
In situ evaluation of tissue. PBS control pig Gp4-4-2021 had no intestinal lesions (**A**). *C. difficile* infected pig Gp4-7-2021 euthanized at PID 5 developed mesocolonic edema and discoloration (**B**). *C. difficile* infected pigs Gp4-9-2021 and Gp4-8-2021 euthanized at PID 10 developed the most severe intestinal lesions of all Gn pigs, including intestinal dilation and inflammation, mucosal hemorrhages, and extensive mesocolonic edema (**C**, **D**)

Upon in situ evaluation, PBS control pigs had no notable intestinal lesions (Fig. 4A). In comparison, all of the *C. difficile*-infected pigs had significant changes to their gastrointestinal tract, with the most severe changes seen in the two pigs euthanized at PID 10. Grossly, the mesocolon of the spiral colon and throughout was moderately to severely distended by clear edema fluid (Fig. 4B–D). Colonic mucosal hemorrhages extending from the ileocecal junction to the rectum were visible in Gp4-8-2021, euthanized at PID 10 (Fig. 4D). Pleural effusion, a systemic complication in severe CDI, was collected from both pigs euthanized at PID 10 (Gp4-8-2021 and Gp4-9-2021).

**Fig. 5:**
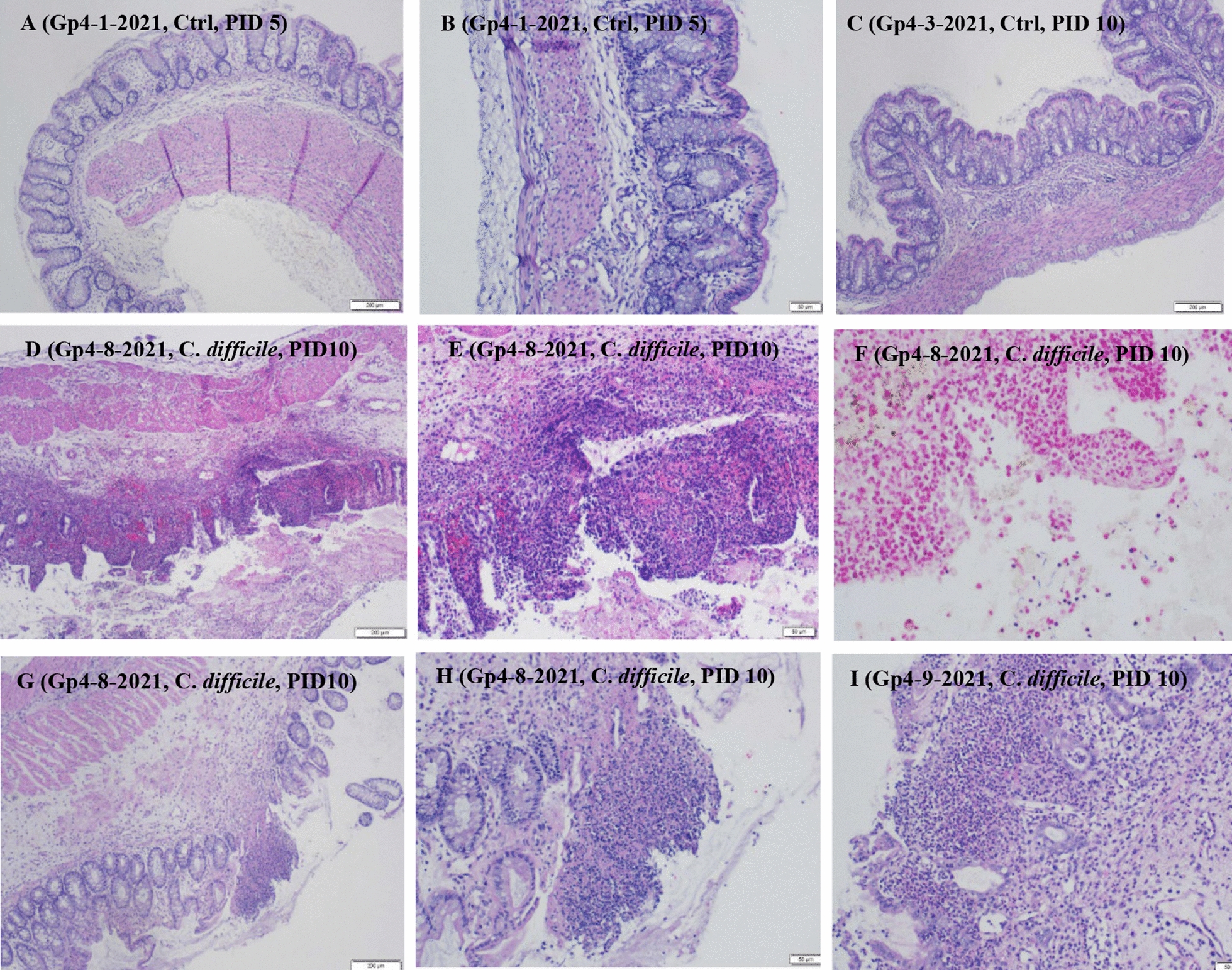
Histological changes in tissue. H&E (**A**–**E**, **G**–**I**) and Gram (**F**) staining of the cecum and colon to detect microscopic lesions induced by *C. difficile*. PBS control pigs had mild edema in the submucosa and lamina propria, however, this did not impact the integrity of epithelial tissue layers (**A**–**C**). Ulceration of the colonic mucosa with replacement by neutrophilic inflammation, fibrin, and necrotic cellular debris (**D**). Greater magnification of the volcano lesion in Gp4-8-2021 (**G**). Gram positive bacterial rods present in the luminal spaces of Gp4-8–2021 tissue (**F**). Severe edema causing substantial expansion of the submucosa and lamina propria, with focal volcano lesion (**G**). Ulceration of the colonic mucosa with accumulation of neutrophils, fibrin, and necrotic debris (**H**, **I**). Scale for images is 200 µm for **A**, **C**, **D**; 50 µm for **B**, **E**, **H**, **I**; 20 µm for **F**

Histological evaluation of sections of the cecum and colon was performed for all pigs. In PBS control pigs, there was evidence of mild edema in the submucosa and lamina propria of the cecum (Fig. 5A–C). While the edema caused expansion of the respective areas, it did not damage nearby blood vessels or disrupt the structural integrity of epithelial tissue layers. Only one PBS control pig had colonic lesions which were extremely mild, consisting of only mild edema in the submucosa and lamina propria. In both the cecum and colon of *C. difficile-*infected pigs, there was severe edema in the submucosa and lamina propria, causing these regions to expand 3–4 times their normal size (Fig. 5D–I). Fluid accumulation was the only noteworthy lesion observed in *C. difficile*-infected pigs at PID 5; however, tissues from PID 10 pigs had significant histological changes. So-called “volcano lesions”, named for the eruption of inflammatory and necrotic cell debris into luminal spaces, are characteristic of severe CDI, and these lesions were observed in both PID 10 pigs (Fig. 5D). Luminal spaces were filled with a combination of neutrophils, fibrin, bacteria, and necrotic debris (Fig. 5E–I). Mucosal lesions were detected in both pigs and ranged from severe erosions to total loss of mucosal epithelium. Glandular structures were intact, but the epithelial lining of these structures was thinner than normal, and the glands were filled with neutrophils and various cellular debris. The spore-forming rods of *C. difficile* bacterial cells were visible in the superficial lamina propria and intraluminal inflammation (Fig. 5F).

### Evaluation of pro-inflammatory cytokines

The concentration of pro-inflammatory cytokines in serum, large intestinal contents (LIC), and pleural effusion was measured by the Porcine Cytokine 13-Plex Discovery Assay. Thirteen cytokines were tested including granulocyte–macrophage colony stimulating factor (GM-CSF), tumor necrosis factor (TNF)-α, interferon (IFN)-γ, Interleukin (IL)-1α, IL-1β, IL-1ra, IL-2, IL-4, IL-6, IL-8, IL10, IL-12, and IL-18. For serum and LIC samples, the fold change between *C. difficile* and PBS control pigs is presented in Table 3. In general, *C. difficile-*infected pigs had higher concentrations of pro-inflammatory cytokines relative to PBS controls pigs. At PID 5, there were increases in all cytokines tested in serum and LIC. The highest fold change in serum was seen in IL-1ra (~ 78). In LIC, the greatest increases in fold change were in IL-1α (~ 63) and TNF-α (~ 21). At PID 10, concentration increases were seen in all but 3 cytokines in serum samples and all but 5 cytokines in LIC samples. IL-1ra again had the highest increase in serum samples (~ 1130-fold) while in LIC IL-1α showed the greatest increase (~ 39,050-fold). The fold change for most cytokines was greater at PID 10 compared to PID 5, reflecting the progression of the disease (Table 3). In the two pleural effusion samples, the cytokines with the highest concentrations were IL-1ra (40,735.95 pg/ml for Gp4-8-2021, 9016.76 pg/ml for Gp4-9-2021) and IL-18 (3237.45 pg/ml for Gp4-8-2021 and 2764.82 pg/ml for Gp4-9-2021) (Table 4).

**Table 3:** Summary of cytokine fold changes in serum and LIC

	Fold changes in cytokine concentration^a^			
	PID5		PID10	
	Serum	LIC	Serum	LIC
GM-CSF	0.88	2.24	1.08	0.66
TNF alpha	1.16	21.09	1.29	13.88
IFN gamma	1.18	2.07	0.77	0.03
IL-1 alpha	1.08	63.29	11.16	3950.62
IL-1 beta	0.89	2.12	18.44	951.98
IL-1 ra	78.02	2.67	1130.69	293.23
IL-2	0.96	1.67	1.05	0.01
IL-4	0.96	5.07	0.87	0.33
IL-6	2.27	0.57	170.92	4.52
IL-8	2.69	10.03	0.89	589.29
IL-10	8.90	2.87	10.01	0.14
IL-12	0.66	8.89	1.16	67.15
IL-18	1.34	1.30	3.47	93.79

^a^The fold change between *C. difficile* and PBS control pigs was calculated by dividing the mean concentration of each cytokine in the *C. difficile* group by the mean concentration in the PBS control group for different sample types on different time points

**Table 4:** Cytokine concentrations (pg/ml) in pleural effusion samples

	Gp4-8-21	Gp4-9-21
GM-CSF	4.55	4.55
TNF alpha	19.94	18.01
IFN gamma	5.69	6.89
IL-1 alpha	11.27	1.62
IL-1 beta	749.96	243.73
IL-1 ra	40,735.95	9016.76
IL-2	1.15	4.57
IL-4	11.45	11.45
IL-6	1357.49	538.03
IL-8	68.82	5.04
IL-10	70.4	19.18
IL-12	194.43	189.47
IL-18	3237.45	2764.82

### Clostridioides difficile colony counting

*C. difficile* colony counting was determined using rectal swabs from PID 0, PID 2, PID 4, and PID 5 (Table 5). As expected, no colonies were detected in samples from PBS control pigs. At PID 2, PID 4, and PID 5, colonies were detected in all *C. difficile* infected pigs.

**Table 5:** *C. difficile* colony counts in rectal swab samples

Gn Pig ID	PBS control				*C. difficile* UK1-inoculated				
	Gp4-1-21	Gp4-2-21	Gp4-3-21	Gp4-4-21	Gp4-5-21	Gp4-6-21	Gp4-7-21	Gp4-8-21	Gp4-9-21
PID0	0	0	0	0	0	0	0	0	0
PID2	0	0	0	0	1.2 × 10^2^	1.68 × 10^4^	1.2 × 10^2^	6.48 × 10^5^	2.88 × 10^4^
PID4	0	0	0	0	0	1.2 × 10^3^	3.96 × 10^4^	1.44 × 10^5^	TMTC
PID5	0	0	0	0	3.72 × 10^4^	1.08 × 10^4^	4.98 × 10^4^	1.2 × 10^3^	1.44 × 10^4^

Numbers of *C. difficile* colonies were measured using rectal swab samples collected at PID 0, PID 2, PID 4, and PID 5. No colonies were detected in the feces of PBS control pigs. *C. difficile* could be detected in the feces of all UK1-inoculated pigs at various points during the study

*TMTC* too many colonies to count

### Cytotoxicity analysis

**Fig. 6:**
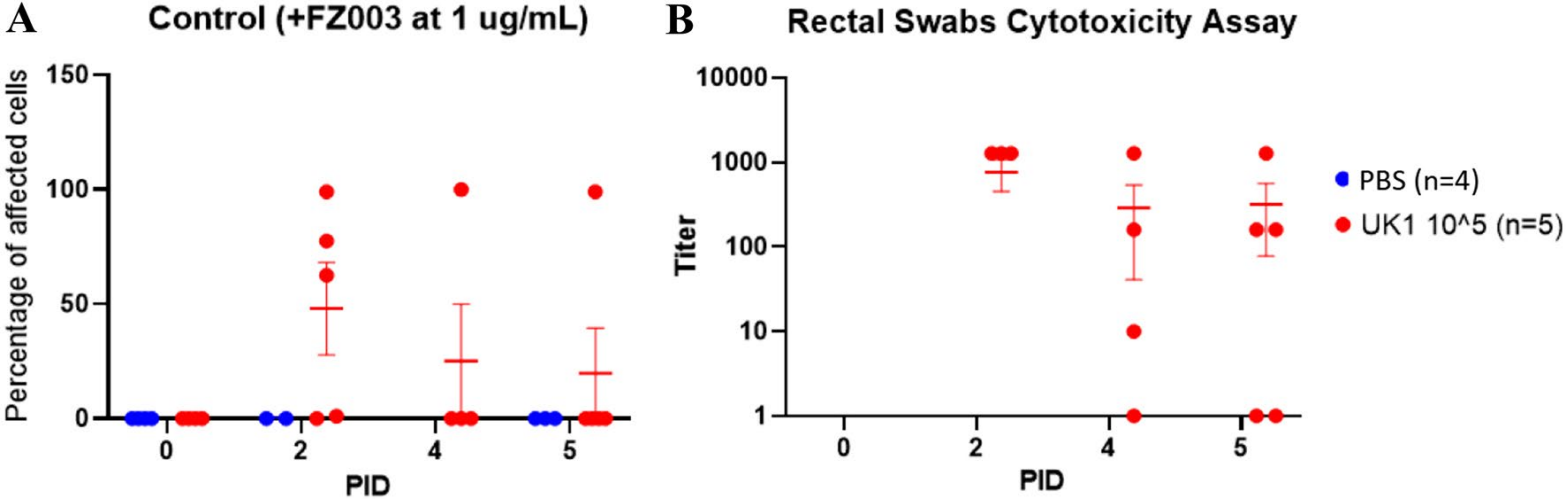
Cytotoxicity assay. *C. difficile* inoculated samples induced cell rounding while PBS control samples had no cytotoxic affect. The neutralizing antibody FZ003 was added to control wells to validate cell rounding was due to *C. difficile* toxins (**A**). The titer is defined as the highest dilution of a sample to cause cell rounding (**B**)

Detection of *C. difficile* toxins in rectal swabs from PID 0, PID 2, PID 4, and PID 5 was performed using cytotoxicity assays (Fig. 6). Toxicity was determined by the percentage of cell rounding following addition of the sample, and the titer was defined as the highest dilution of a sample to cause cell rounding. No cell rounding was observed in cells exposed to PBS control pig samples. *C. difficile*-infected samples at PID 2, PID 4, and PID 5 all caused cell rounding, indicating that a high level of toxin was present. The average toxin titer for *C. difficile*-infected samples was 960, 363, and 400 for PID 2, PID 4, and PID 5 respectively.

## Discussion

*Clostridioides difficile* has a high prevalence in food animals, especially in neonatal animals; on average, the prevalence of *C. difficile* in neonatal piglets is ~ 70% [15]. Affected pigs experience enteritis, decreased weaning weight, and in severe cases, respiratory distress and death [16]. The similarities between the CDI occurring in pigs and humans and the availability of reagents to study porcine immune responses make pigs a more attractive model for CDI [11] than the hamster or mouse model [11, 25].

The present study reports the successful establishment of a Gn pig model of CDI and disease after oral challenge with 1 × 10^5^ spores of UK1 (027/B1/NAP1 hypervirulent strain) [17–19]. UK1 is a clinically relevant strain connected to the increase in numbers of severe CDI acquired in US hospitals [20, 21]. UK1 produces all three toxins and has demonstrated greater resistance to the fluoroquinolone class of antibiotics than other *C. difficile* ribotypes [22]. While mice and Golden Syrian hamsters have historically been used to study aspects of UK1 infection [23, 24], these models have multiple limitations. Hamsters have a different site of infection (cecum versus colon), tend to develop the fulminant disease, and have limited available reagents [11, 25]. Mouse models of CDI have been significantly improved, however, mice require pretreatment with antibiotic cocktails to bypass their natural predisposition to resisting *C. difficile* colonization [11, 14, 25]. Gn pig models can bypass the antibody cocktail pretreatment step due to their naturally microbiota free gut. Additionally, they have human-like digestive and immune physiology and experience a similar infection progression as humans [11–13]. These traits make Gn pigs more advantageous than conventional animal models and would allow researchers to study multiple aspects of CDI including, but not limited to, relapse infections, elucidating the role of CDT in virulence, and standardizing the CDI severity index.

There is significant diversity of CDI presentation in humans. While most patients develop mild diarrhea that persists for 5–10 days, symptom presentation can vary based on a variety of factors including age, prior antibiotic usage, and immunocompromised health status [1]. For example, infants are more likely to be asymptomatic carriers whereas populations ≥ 65 years old are more likely to develop severe CDI with systemic complications [9, 26]. Even within age groups, trends in symptom presentation can vary. Formula-fed infants are at an increased risk of colonization compared to their breast-fed counterparts due to a lack of maternal antibodies received via passive immunization [26]. Our model reflected this diversity as *C. difficile* inoculated piglets displayed diarrhea of mixed severity, impaired weight gain, and large intestinal lesions that ranged from moderate to severe edema, colonic inflammation, and mucosal hemorrhages. One pig (Gp4-8-21) presented with signs of systemic disease seen only in severe CDI, including lethargy, cyanosis, and accumulation of pleural effusion. Development of systemic disease is expected when there is a high titer of toxin circulating in infected animals, and a detailed examination of signs of systemic disease has been reported prior [6].

*Clostridioides difficile* pathophysiology involves direct injury to the intestinal mucosa by the secretion of bacterial enterotoxins that induce host inflammatory responses. When TcdA and TcdB have functional glycosyltransferase activity, they are potent inducers of cytokine responses, which promote neutrophilic activation and recruitment [3]. In human patients, the most upregulated cytokines include IL-1, IL-6, IL-8, IL-16, and IL-17a [3]. Other cytokines known to be stimulated include IL-1β, IL-2, IL-5, IL-10, IL-12, IL-13, IL-15, IL-22, IFN-γ, and TNF-α [3, 27, 28]. Of these, disease severity can be traced to specific upregulation of IL-2, IL-5, IL-15, and IFN-γ. IL-5 and IFN-γ are associated with mild to moderate cases of CDI whereas IL-2 and IL-15 are correlated to severe CDI cases [3]. In our Gn pig model, as in humans, proinflammatory cytokines were upregulated in serum and LIC samples from *C. difficile* inoculated pigs relative to PBS control pigs. There were remarkable increases in the levels of IL-1α, IL-1β and IL-8 in the LIC and IL-1ra in both serum and LIC in Gn pigs tested at PID 10. These same cytokines could be detected in significant quantities in pleural effusion samples taken at the same time point. These cytokine responses indicate activation of the host defense system and correspond to the more severe inflammation seen in the tissues of *C. difficile* inoculated piglets during necropsy and H&E staining.

The role of IL-8 during *C. difficile* infection of Gn pigs has been described previously by Steele et al 2010. IL-8 is a known mediator of neutrophil activation, and dramatic increases are seen in the large intestine of Gn pigs following inoculation with *C. difficile* [2]. Here, we confirm the results and add that increases are also seen in serum and pleural effusion samples of infected pigs. Additionally, we put forward other cytokine markers of the disease that support the model’s resemblance to immune responses observed in humans. During the acute stage of CDI, IL-1β, IL-12, and IL-23 are upregulated and trigger the production of effector cytokines like IL-17a, IL-22, IFN-γ, and TNF-α [28]. In our model, *C. difficile* infected pigs had the expected increases of IL-1β, IL-12, IFN-γ, and TNF-α, indicating activation of the intestinal innate lymphoid cells and the corresponding increase in effector cytokines.

This model will facilitate future research of anti-CDI strategies. First, having an animal model that mimics the profile of cytokine responses occurring in human patients will help to understand the mechanisms behind acute inflammation. This in turn will help to develop new therapeutics, such as antibiotics in combination with anti-inflammatory drugs and monoclonal antibodies, to target specific inflammatory pathways and modulate disease. Second, it is well known that a healthy gut microbiome is key for inhibiting *C. difficile* colonization. One method to counter recurrent CDI is using fecal microbiota transplantation (FMT) to reintroduce healthy gut flora [29]. While FMT has shown promise in early trials, it requires more studies to standardize the practice [29]. Following oral inoculation with fecal suspensions from children, Gn pigs have the ability to reproduce a flourishing gut microbiome similar to what is seen in humans [30]. This would make them ideal for FMT trials to determine which components are most protective against primary and secondary *C. difficile* infection.

## Conclusion

In this study, we successfully established a model of *C. difficile* infection and disease in Gn pigs. The Gn pig model is highly applicable to many aspects of *C. difficile* research and will be a key part of our future work evaluating the effectiveness of our engineered probiotic yeast immunotherapy against *C. difficile* [19]. Neutralizing antibodies have been shown to play a role in mitigating toxin-induced disease, and early animal studies with this kind of therapeutic have shown success in preventing the development of systemic CDI [6, 31]. Other potential applications of this model include profiling the immune response following infection and standardizing the usage of FMT. Gn pigs are an invaluable resource for the *C. difficile* research community and are sure to be a springboard for new discoveries in the years to come.

## Data Availability

All data are included in this manuscript.

## Abbreviations

Gn: Gnotobiotic
*C. difficile*: *Clostridioides difficile*
CDI: *Clostridioides difficile* Infection
TcdA: Toxin A
TcdB: Toxin B
CDT: *Clostridioides difficile* Binary toxin
FMT: Fecal microbiota transplantation
PID: Post inoculation day
LIC: Large intestinal contents
